# Response to change in the number of visual stimuli in zebrafish:A behavioural and molecular study

**DOI:** 10.1038/s41598-020-62608-5

**Published:** 2020-04-01

**Authors:** Andrea Messina, Davide Potrich, Ilaria Schiona, Valeria Anna Sovrano, Scott E. Fraser, Caroline H. Brennan, Giorgio Vallortigara

**Affiliations:** 1 Center for Mind/Brain Sciences, University of Trento, Rovereto, Italy; 2 Department of Psychology and Cognitive Science, University of Trento, Rovereto, Italy; 3 Michelson Center for Convergent Bioscience, University of Southern California, Los Angeles, USA; 4 School of Biological and Chemical Sciences, Queen Mary University, London, UK

**Keywords:** Molecular biology, Cognitive neuroscience

## Abstract

Evidence has shown that a variety of vertebrates, including fish, can discriminate collections of visual items on the basis of their numerousness using an evolutionarily conserved system for approximating numerical magnitude (the so-called Approximate Number System, ANS). Here we combine a habituation/dishabituation behavioural task with molecular biology assays to start investigating the neural bases of the ANS in zebrafish. Separate groups of zebrafish underwent a habituation phase with a set of 3 or 9 small red dots, associated with a food reward. The dots changed in size, position and density from trial to trial but maintained their numerousness, and the overall areas of the stimuli was kept constant. During the subsequent dishabituation test, zebrafish faced a change *(i)* in number (from 3 to 9 or *vice versa* with the same overall surface), or *(ii)* in shape (with the same overall surface and number), or *(iii)* in size (with the same shape and number). A control group of zebrafish was shown the same stimuli as during the habituation. RT-qPCR revealed that the telencephalon and thalamus were characterized by the most consistent modulation of the expression of the immediate early genes *c-fos* and *egr-1* upon change in numerousness; in contrast, the retina and optic tectum responded mainly to changes in stimulus size.

## Introduction

Numerical abilities can be apparent in association with a symbolic and a non-symbolic system^1–3^. The former is a human-specific trait, which supports precise numerical determination through the use of symbols (e.g. Arabic or Roman numerals) belonging to a cultural tradition. The latter is a language-independent, non-symbolic mechanism that exploits magnitude and approximation for representing numerical sets of physical elements in an analogue fashion, the so-called Approximate Number system (ANS).

The ANS supports approximate discrimination between sets of items of different numerousness with a degree of accuracy, which is ratio-dependent, i.e. following Weber’s Law^4–7^. The existence of another, though non-quantitative, system to deal with the individuation of small numerosities on the basis of working memory alone, and thus with an upper limit of about 3 items (the so-called Object File System), has been claimed for but evidence in non-human animals is currently disputed^8,9^.

The ability to evaluate numerical information and compare quantities is thought to represent an ecological advantage in the interactions between organisms and their surrounding environment. Animals exploit this ability in order to optimize foraging decisions^10^, responses to aggressive behaviours^11,12^, defence against predators or to predate efficiently^13,14^, and to estimate the number of social companions^15–17^. Numerical abilities associated with the ANS have been documented in a variety of species, including mammals^10,18^, amphibians^19,20^, reptiles^21,22^, birds^23,24^, fishes^15,17,25^ and insects^26,27^.

Even though behavioural evidence for numerical abilities associated with the ANS are widespread in non-human animals, data concerning their neural bases are confined to non-human primates and corvids and to the use of single cell recordings^28,29^. Nieder and collaborators discovered populations of neurons selectively sensitive to number in the endbrain of numerically-naive corvids^30,31^ and in the intraparietal sulcus (IPS) and dorsolateral prefrontal cortex (PFC) of non-human primates^32^. A role for the posterior part of the parietal cortex in number cognition has been documented in humans using fMRI techniques^33^.

Most of the species for which there is evidence for numerical (quantity) cognition do not possess, however, a cortex (e.g. fish, amphibians, reptiles). In birds, in which pallial regions equivalent to mammalian cortex have been proposed - arranged in aggregates rather than laminae^34,35^ - it seems that the endbrain regions in which neurons responding to numerousness have been discovered could be equivalent (though not homologous) to the mammalian prefrontal areas. Interestingly, there is evidence that, even in humans, subcortical regions can be crucially involved in response to numerousness^36^.

Numerical abilities in fish have been extensively studied with a variety of different behavioural paradigms, such as spontaneous choice for different numerousness of social companions^16,17,37,38^, items of food^17,25^ or using operant conditioning^17^. Although the ability to perform numerical tasks has been reported in several fish species, the underlying neural mechanisms remain unknown.

Zebrafish (*Danio rerio*) also possess numerical abilities^16,39^, and, given their extensive use as a model system in biology^40–44^, they are ideally suited for an investigation of the molecular mechanisms of ANS. Among the most used methods for investigating the neural bases of behaviour in zebrafish^45^, are the immediate early genes (IEGs), which consist of a limited number of genes that have the capacity to quickly respond to regulatory signals^46,47^. They are expressed transiently (usually lasting a few hours) and very quickly upon both cell-intrinsic and cell-extrinsic stimuli^47^ linked to different pathways. IEGs mRNA is usually transcribed within minutes following specific signals^48^ and, thanks to such characteristics - despite their downstream targets and their function have yet to be fully unveiled^49^ - IEGs have been widely used as markers of neuron activity^50^.

As a first step, we aimed to identify the brain regions that are involved in quantity discrimination processes, using the expression of the IEGs *c-fos* and *egr-1*, which are widely employed as transient markers of neuronal activity^49^ using a response to novelty, habituation/dishabituation paradigm. During the habituation phase, we repeatedly presented zebrafish with a set of visual elements (small dots; either 3 dots or 9 dots) controlled for continuous physical variables (surface area, position, density); subsequently, during the dishabituation phase, a novel stimulus was shown to the fish. Separate groups of fish where presented with a change in *(i)* numerousness (a different number of items compared to the habituation phase, maintaining the same overall surface); *(ii)* shape (a different shape, with the same overall surface and numerousness); or *(iii)* size (a larger or a smaller overall surface area, with the same shape and numerousness). Control fish were shown the same stimulus as during the habituation phase. Thirty minutes after the dishabituation test, zebrafish were sacrificed, their brains were dissected and processed, and the expression of c-fos and egr-1 was investigated in order to identify those regions with changes in neuronal activation to identify the potential neural correlates associated with changes in quantity (number, size) or shape.

Since the neuronal and molecular bases of numerousness in zebrafish are totally unknown, we decided to focus as a first step on the major brain regions of the vertebrate brain: retina, optic tectum, thalamus, telencephalon, cerebellum and medulla oblongata.

## Results

### Behaviour

**Figure 1:**
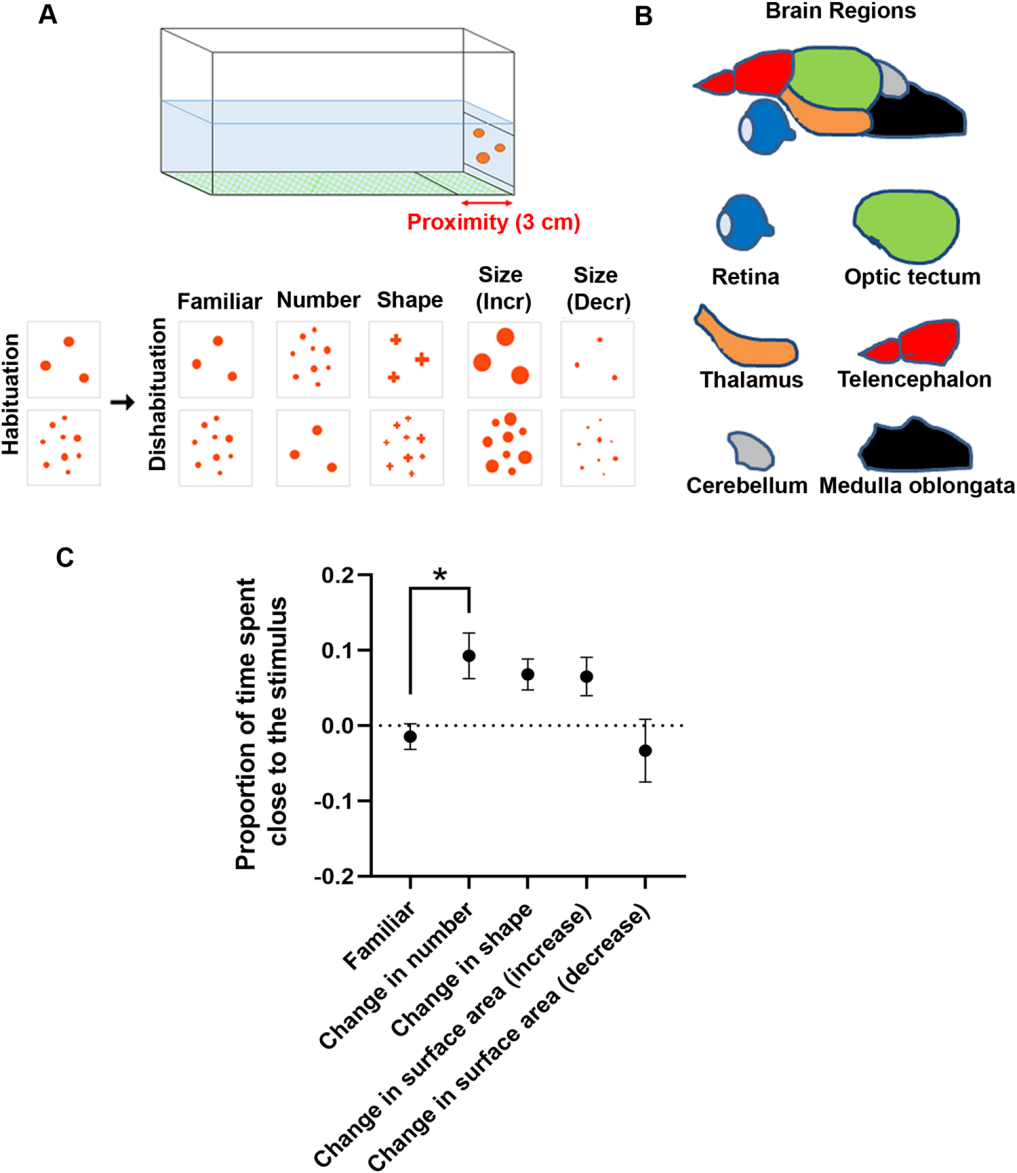
(**A**) Apparatus and stimuli used for the habituation and dishabituation phases. (**B**) Brain regions exploited for molecular biology analyses: retina, optic tectum, thalamus, telencephalon, cerebellum, medulla oblongata. (**C**) Dishabituation results expressed as proportion of time spent near the stimulus (comparison between dishabituation and habituation trials) in the different testing conditions (*p < 0.05, Dunn’s post hoc tests, with Bonferroni correction). Group means with SEM are shown.

Data concerning the proportion of time spent near the stimulus (comparing the dishabituation trial with the first of the four habituation trials previously performed) were analyzed with a Kruskal-Wallis test (variances were not homogenous) with habituation (3 or 9 elements) and test (no change (familiar), change in number, change in shape, change in surface area (increase), change in surface area (decrease)). There was a significant effect of test (χ^2^ (4) = 15.518, *p* = 0.004) but not of habituation (χ^2^ (1) = 0.494, *p* = 0.482.). The results are shown in Fig. 1, collapsed for the two habituation conditions -i.e., habituation with 3 and 9 dots- are considered together because no significant difference between the two conditions was observed (above); separate graphs for the two conditions are however shown in the Supplementary Materials, see Fig. 1 Supplementary Materials.

Dunn’s post hoc tests (with Bonferroni correction) revealed a significant difference only between the control (familiar) condition and the change in number (*p* = 0.021), but not for the other types of changes (shape: *p* = 0.088; surface area (increase): *p* = 0.315; surface area (decrease): *p* > 0.5).

### Immediate early gene (IEG) expression

Due to their different expression pathways, separate analyses of variance (ANOVA) were performed for *c-fos*^48,51^ and for *egr-1*^48,52^, with habituation (habituation with 3 dots, habituation with 9 dots) and type of test [familiar (control condition with no change), number, shape, surface area increase, surface area decrease] as between-subject factors, and brain areas (retina, optic tectum, thalamus, telencephalon, cerebellum, medulla oblongata) as a within subject factor.

The complete outcome of the oneway ANOVAs is shown in the Supplementary Materials (Supplementary Materials Table 1). Given that interactions involving brain areas, test and habituation were significant for both *c-fos* (*F*(16.885, 253.272) = 3.228, *p* = 0.0001) and *egr-1* (*F*(13.3335, 200.028) = 1.934, *p* = 0.027), in subsequent analyses we considered habituation and test separately for the different brain areas.

#### Retina

The complete ANOVAs for *c-fos* and *egr-1* are shown in the Supplementary Materials (Supplementary Materials Table 2).

**Figure 2:**
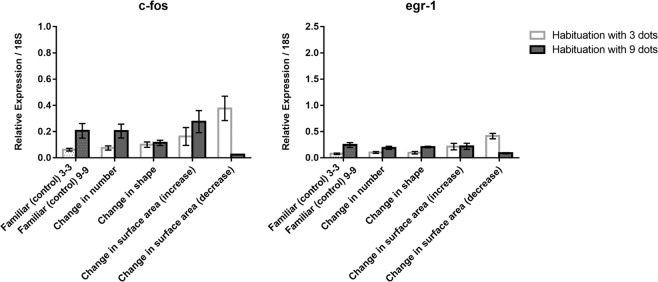
Relative expression of *c-fos* and *egr-1* IEGs in the retina in the different test conditions; group means with SEM are shown.

As to *c-fos* (Fig. 2 top) a significant interaction between habituation and test was observed (*F*(4, 60) = 7.929, *p* = 0.0001), whereas the main effects were not significant. As can be seen in Fig. 2 the familiar, number and shape conditions only revealed a significant effect of habituation (*F*(1, 36) = 11.460, *p* = 0.002), with more *c-fos* expression in the group habituated with the larger number of dots. The interaction between habituation and test was confined to the change in surface areas conditions (*F*(2, 36) = 9.868, *p* = 0.0001). Comparing familiar *vs*. surface area change revealed a significantly higher *c-fos* expression as a result of the increase in size (*F*(1, 24) = 4.407, *p* = 0.046) irrespective of habituation conditions, whereas in the case of the decrease in size there was higher *c-fos* expression following habituation to 3 dots and lower expression following habituation with 9 dots (*F*(1, 24) = 20.918, *p* = 0.0001).

As to *egr-1* (Fig. 2 bottom), the ANOVAs revealed only a significant effect of test (*F*(4, 60) = 2.981, *p* = 0.026) and of the interaction between test and habituation (*F*(4, 60) = 13.541, *p* = 0.0001). Again, the interaction was due to the changes in surface area conditions. An analysis restricted to only familiar, number and shape (see Fig. 2, bottom graphs) revealed only a main effect of habituation (*F*(1, 36) = 31.548, *p* = 0.0001) with a general higher activation with 9 dots. The increase in surface area produced no significant overall effects in *egr-1* expression (*F*(1, 24) = 2.729, *p* = 0.112), whereas the decrease in surface area produced an increase in *egr-1* expression in fish habituated with 3 dots and a decrease in fish habituated to 9 dots (p = 0.01 Tukey test Bonferroni corrected for multiple comparison).

Overall, it appeared that in the retina only changes in surface areas affected immediate early gene expression in a way that was modulated by habituation conditions.

#### Optic tectum

The complete ANOVAs for c-fos and egr-1 are shown in the Supplementary Materials (Supplementary Materials Table 3).

**Figure 3:**
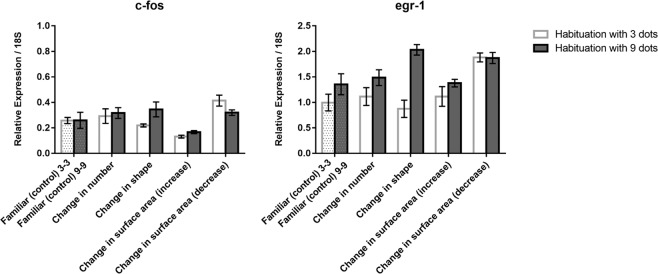
Relative expression of *c-fos* and *egr-1* IEGs in the optic tectum in the different test conditions; group means with SEM are shown.

As to c-fos (Fig. 3 top), the ANOVA revealed only a significant main effect of test (*F*(4, 60) = 8.410, *p* = 0.0001). This effect was however only due to the changes in surface areas for an analysis restricted to familiar, number and shape did not show any significant heterogeneity (*F*(2, 36) = 0.169, *p* = 0.854). Comparing familiar (control) condition with changes in surface area revealed a decrease of c*-fos* expression when the area was increased (*F*(1, 24) = 11.735, *p* = 0.002) and an increase of *c-fos* expression when the area was decreased (*F*(1, 24) = 6.420, *p* = 0.018).

Expression of *egr-1* (Fig. 3 bottom) revealed a more complex pattern because change in shape was also affected. significant effects of test (*F*(4, 60) = 6.948, *p* = 0.0001), habituation (*F*(1, 60) = 20.291, *p* = 0.0001) and habituation x test: (*F*(4, 60) = 4.243, *p* = 0.004). There was increased *egr-1* mRNA expression in fish habituated with 9 dots (but not in those habituated with 3 dots) when comparing familiar *vs*. shape (test x habituation: *F*(1, 24) = 5.887, *p* = 0.023). The comparison between familiar and change of size revealed that the decrease in surface area resulted in an increase of *egr-1* expression (*F*(1, 24) = 22.023, *p* = 0.0001), whereas the increase in surface area did not produce any effect.

Even in the optic tectum immediate early gene expression was affected only by change in surface area and, limited to egr-1, to changes in shape but in a way that appeared to be modulated by habituation/dishabituation conditions.

#### Thalamus

The complete ANOVAs for c-fos and egr-1 are shown in the Supplementary Materials (Supplementary Materials Table 4).

**Figure 4:**
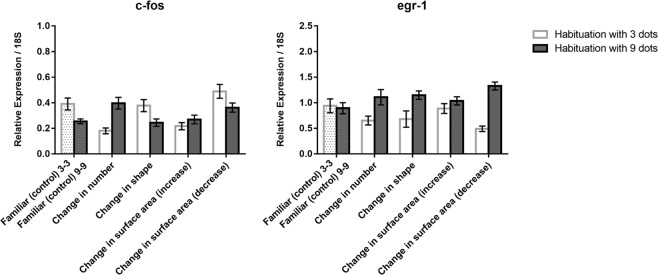
Relative expression of *c-fos* and *egr-1* IEGs in the thalamus in the different test conditions; group means with SEM are shown.

In the thalamus (Fig. 4 top), the analysis for *c-fos* revealed only significant effects of test (*F*(4, 60) = 6.329, *p* = 0.0001) and habituation x test (*F*(4, 60) = 8.629, *p* = 0.0001). A comparison between familiar and number revealed a decrease of *c-fos* mRNA levels in fish habituated with 3 dots (LSD test, *p* = 0.001) and an increase in fish habituated with 9 dots (LSD test, *p* = 0.006). In contrast, the change in shape revealed only a main effect of habituation (*F*(1, 24) = 12.953, *p* = 0.001) when compared to familiar condition but no effects of test. The increase in surface area resulted in a main effect of test (*F*(1, 24) = 5.567, *p* = 0.027) and of habituation x test (*F*(1, 24) = 7.926, *p* = 0.011), with a decrease in activation following habituation with 3 dots (and test with 9 dots) and an increase following habituation with 9 dots (and test with 3 dots). The decrease in surface area did not show any test x habituation interaction (*F*(1, 24) = 0.011): *c-fos* activation increased with respect to controls (Familiar) irrespective of habituation conditions.

The analysis of *egr-1* mRNA (Fig. 4 bottom) revealed a significant effect of the habituation (*F*(1, 60) = 11.356, *p* = 0.001) and test (*F*(4, 60) = 4.369, *p* = 0.004), but not of the interaction habituation x test (*F*(4, 60) = 2.190, *p* = 0.081). An ANOVA restricted to familiar, number and shape revealed a significant effect only of habituation (*F*(1, 36) = 8.303, *p* = 0.007), with higher activation following activation with 9 dots. The increase in surface area did not reveal any significant effect. In contrast, the decrease in surface area was associated with increased activation of egr-1. (*F*(1, 24) = 13.157, *p* = 0.001).

The thalamus, differently than previous areas, first revealed c-fos responsivity to number, with a decrease of *c-fos* mRNA levels in fish habituated with 3 dots and then tested with 9 dots and an increase in fish habituated with 9 and tested with 3 dots. The results were similar for egr-1 but the interaction between test and habituation failed to reach the conventional level of statistically significant (see above, *p* = 0.081). This pattern cannot easily be accommodated with the simple idea that an increase in the number of stimulus elements would be associated with more cells activated (actually, the reverse was observed). Sensitivity to changes in shape was not observed, whereas a sensitivity to changes in size persisted, still modulated by the direction of change (increase or decrease in surface areas) and habituation conditions.

#### Telencephalon

The complete ANOVAs for c-fos and egr-1 are shown in the Supplementary Materials (Supplementary Materials Table 5).

**Figure 5:**
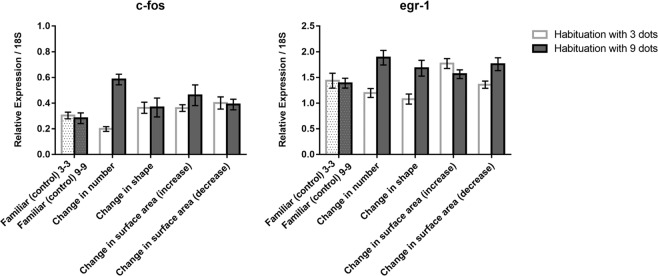
Relative expression of c-fos and egr-1 IEGs in the telencephalon in the different test conditions; group means with SEM are shown.

In the telencephalon (Fig. 5 top), a comparison between familiar (control) and number revealed an effect of habituation (*F*(1, 24) = 24.991, *p* = 0.0001), test (*F*(1, 24) = 11.044, *p* = 0.003) and the habituation x test interaction (*F*(1, 24) = 31.748, *p* = 0.0001), with a decrease of *c-fos* mRNA levels in fish habituated with 3 dots (LSD test, *p* = 0.057) and an increase in fish habituated with 9 dots (LSD test, *p* = 0.001).

In contrast, an ANOVA comparing familiar (control) with shape and surface area did not reveal any significant effect (habituation: *F*(1, 48) = 0.223, *p* = 0.639; test: *F*(3, 48) = 2.121, *p* = 0.110; habituation x test interaction: *F*(3, 48) = 0.598, *p* = 0.619).

Similar results were obtained for *egr-1* (Fig. 5 bottom) as to number: a decrease of *egr-1* mRNA levels was observed in fish habituated with 3 dots (LSD test *p* = 0.050) and an increase in those habituated with 9 dots (LSD test *p* = 0.001). Differently than c-fos expression, significant increase of *egr-1* was detected also for the increase of surface area in zebrafish habituated with 3 dots (*p* = 0.027) and for the decrease of surface area in zebrafish habituated with 9 dots (*p* = 0.042).

In the telencephalon there was the same pattern of selectivity to change in numerosity observed in the thalamus. Immediate early gene expression increased with decreased numerosity and *vice versa*. Selectivity to shape and surface areas were absent or confined to only surface area by egr-1 expression and modulated by direction of change and habituation.

#### Cerebellum

The complete ANOVAs for c-fos and egr-1 are shown in the Supplementary Materials (Supplementary Materials Table 6).

**Figure 6:**
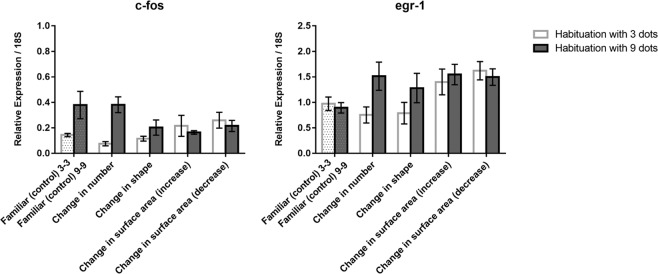
Relative expression of *c-fos* and *egr-1* IEGs in the cerebellum in the different test conditions; group means with SEM are shown.

In the cerebellum (Fig. 6 top), for *c-fos* a main effect of habituation (*F*(1, 60) = 8.713, *p* = 0.005) and habituation x test (*F*(4, 60) = 4.037, *p* = 0.006), but not a main effect of test (*F*(4, 60) = 1.025, *p* = 0.402) were observed. A comparison confined to familiar, number and shape showed an effect only of habituation, with higher expression in animals habituated to 9 dots (*F*(1, 36) = 20.077, *p* = 0.0001). The change in surface area revealed only a minor habituation x test interaction (*F*(2, 36) = 3.310, *p* = 0.048).

As to *egr-1* (Fig. 6 bottom), the ANOVA revealed only a main effect of test (*F*(4, 60) = 3.616, *p* = 0.010). A significant increase of *egr-1* expression was detected in the comparison between the familiar (control) condition and both the increase (LSD test, *p* = 0.014) and decrease (LSD test, *p* = 0.005) of surface areas conditions.

Basically, immediate early gene expression in the cerebellum was affected only by changes in surface areas and confined to *egr-1*.

#### Medulla oblongata

The complete ANOVAs for c-fos and egr-1 are shown in the Supplementary Materials (Supplementary Materials Table 7).

**Figure 7:**
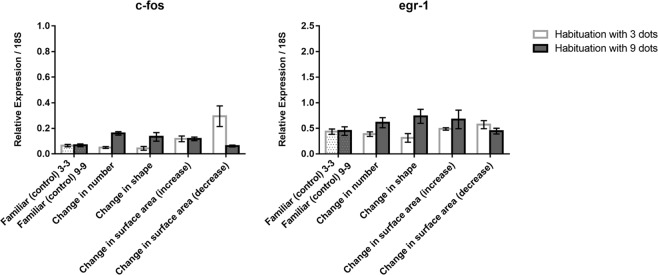
Relative expression of *c-fos* and *egr-1* IEGs in the medulla oblongata in the different test conditions; group means with SEM are shown.

In the medulla oblongata (Fig. 7 top), a general ANOVA for *c-fos* revealed a significant effect of test (*F*(4, 60) = 3.956, *p* = 0.006) and habituation x test (*F*(4, 60) = 10.348, *p* = 0.0001). A significant increase of *c-fos* expression levels was observed for the decrease in surface area condition in fish habituated with 3 dots (*p* = 0.0001), whereas an increase of *c-fos* was apparent in number (*p* = 0.001) and in shape (*p* = 0.016) change conditions but only in fish habituated with 9 dots.

As to *egr-1*, there was only a main effect of the habituation (*F*(1, 60) = 5.657, *p* = 0.021) but not of test (*F*(4, 60) = 0.561, *p* = 0.692). The main effect reveals a general trend for higher expression in fish habituated to 9 dots.

Immediate early gene expression in the medulla oblongata did not reveal a clear pattern. Modulation by change in surface area, number and shape were dependent on habituation for *c-fos* and absent for *egr-1*.

Taken together, our data suggest that the zebrafish thalamus, telencephalon and, to some very limited extent, medulla oblongata, are involved in the elaboration of numerical stimuli and in quantity estimation. In contrast, the retina, the optic tectum and the cerebellum appear to be primarily in charge of processing the change in the overall surface area of the stimuli.

## Discussion

Our research study was focused on designing a protocol that took advantage of a behavioural paradigm of habituation/dishabituation followed by molecular biology assays in order to start investigating the neuronal bases of quantity (both discrete and continuous) discrimination. We chose zebrafish as an animal model, since it has been demonstrated that teleost fish possess numerical abilities^16,17^.

Experimental protocols similar to the one exploited in this work have been previously applied to investigate numerical abilities in chicks^24^ but have not been used in fish species.

In the behavioural tests we found that zebrafish showed a general increase in approach when exposed to a novel stimulus with respect to the familiar condition, irrespective of the habituation stimuli (3 or 9 dots). However, because of the variability in the data, a significant effect in behaviour was noticed only for a change in numerousness. This is interesting, however, because it confirms evidence that numerousness is consistently used by non-human animals, even more so than changes in stimulus parameters (like surface area), which (usually) co-vary with change in numerousness^53^.

In the second part of the experimental protocol, fish were sacrificed and their brains were dissected in order to study the brain regions of the zebrafish involved in the ability to discriminate quantities, via the analysis of the expression of immediate early genes (IEGs) by RT-qPCR. IEGs have been widely used as markers of neuronal cell activation upon specific stimuli and their expression is correlated to the number of activated cells^54^.

In the retina, IEG expression was modulated only in response to a change in surface area; and the same was observed in the tectum, where for instance a decrease of *c-fos* expression was associated with the increase in surface area and an increase of *c-fos* expression with the decrease in surface area. Data for IEG modulation by changes in surface area in the tectum fit with evidence for neurons in this area tuned to the size of the stimuli^55^.

The thalamus and the telencephalon showed a similar pattern but for number rather than for surface area, with a decrease of *c-fos* mRNA levels in fish habituated with 3 dots (and tested for novelty with 9 dots) and an increase in fish habituated with 9 dots (and tested for novelty with 3 dots). Changes associated with shape or surface area were less clear or absent.

Even for the cerebellum and medulla oblongata, changes, when observed, were limited to surface area rather than number, or confined to specific interactions with habituation conditions.

Intriguingly, depending on the habituation stimulus, we noticed an opposite trend in the modulation of the IEGs upon the change of numerosity in the thalamus and telencephalon (and a somewhat similar pattern was observed for size in the tectum, at least for *c-fos*). This pattern of modulation is compatible with either one of two different models. It could be that the gene expression modulation that we observed - e.g. in the telencephalon for number - could be due to the activation of single unimodal neurons that, by summating their inputs, activate the numerousness cluster corresponding to the presented numerosity^56^; or alternatively that it could be due to the signal integration of the dendrites of specific number detector neurons^57^. Further analyses will be necessary to explore single unimodal or signal integration nature of, if existing, number neurons in the zebrafish brain. For example, the precise location and cell body of numerousness-responding neurons will need to be identified and validated in the zebrafish telencephalon using microdissection or histological procedures and pharmacological or genetic functional validation. Moreover, establishing specific inducible fluorescent transgenic lines in order to target number-selective neurons will also appear necessary.

Taken together, our results suggest that the telencephalon and the thalamus could be the major regions involved in number discrimination in zebrafish. These results are in agreement with findings in others vertebrate species, including humans. In fact, telencephalic areas linked to (approximate) number representation have been discovered in non-human primates and in corvids^58^. Indeed, despite their distinct evolutionary pathways, which resulted in dissimilar endbrains, corvids and monkeys are characterized by advanced cognitive abilities and both groups attend to numerical information via the ANS. The presence of number-responsive neurons in the intraparietal sulcus (IPS)^59^ and dorsolateral prefrontal cortex (PFC)^60^ of monkeys and in the nidopallium caudolaterale (NCL) of corvids has been well documented^58^. Additionally, the thalamus has been linked to numerosity in humans. A study conducted by Kovas and collaborators^59^, for example, investigated the brain areas associated with number estimation in human infants using fMRI, highlighting the thalamic region as one of the activated areas.

Since our RT-qPCR results seem to support the existence of neural mechanisms associated with numerousness in the telencephalon and thalamus, and of mechanisms associated with continuous quantity (surface area) in the optic tectum and retina, it will be important to perform immunohistochemical and *in-situ* hybridization assays, which would provide more detailed data in relation to these abilities in zebrafish. Also, further analyses within such large areas such as the telencephalon will be needed in order to identify specific sub-regions associated with numerousness representation.

## Materials and Methods

### Animals

We used 120 nine-month-old wild type male zebrafish, *Danio rerio*, housed in our laboratory in an automated aquarium system (ZebTEC Benchtop, Tecniplast), with 3.5-litre plastic tanks. Animals were divided in sex groups of 10 individuals and reared in standard conditions (28 °C, light/dark cycle of 12 h/12 h, fed three times a day with dry food) in accordance with the guidelines of animal welfare.

### Habituation-dishabituation test

#### Apparatus and stimuli

The behavioural experimental setup consisted of a rectangular tank (40 × 60 × 30 cm) made of a white plastic material (poliplak) on the four sides, with a green mesh (grid 0.4 mm thick) forming the base of the tank. A group of five tanks was placed in a larger white plastic arena (20 × 6.5 × 20 cm), raised 15 cm from the base of the arena. Since fish were left singly in the tanks for the whole experiment (lasting 5 days), the mesh tank bottom allowed for good water circulation, letting uneaten food and excrement pass through and be removed by a pump and a filter system (Micro Jet Filter MCF 40).The water was kept at a constant temperature of 26 °C and its level in each tank was 8 cm. The apparatus was lit by 2 led stripes and a webcam (Microsoft LifeCam Studio) recorded fish behaviour from above (50 cm) the setup.

The stimuli used for the habituation and dishabituation phases were laminated cards (6 × 6 cm) glued on white plastic panels (20 × 6 cm). During each trial, the stimulus panel was manually inserted into the experimental tank in proximity of one of the two shorter sides.

During the habituation phase, the stimuli consisted of 3 or 9 red/orange (RGB:252,72,11) dots on a white background. For each numerosity, a set of 9 different stimuli configurations was created, randomizing the spatial distribution as well as the size of each dot (range of the dots’ size was between 4 and 11 mm). The total cumulative surface area of the stimuli (1.58 cm^2^) was equalized among the different stimuli configurations.

For the dishabituation phase, new sets of nine novel stimuli were used. Depending on the dishabituation condition, the novel stimulus could change from the habituation phase *(i)* in number (from 3 to 9 dots or *vice versa*, maintaining the overall area), *(ii)* in shape (from dots to squares, keeping the number and the overall surface area unchanged) or *(iii)* in size (increasing or decreasing three times the overall dot surface area, but keeping the same shape and number). Similarly to the habituation phase, in each dishabituation test the spatial distribution and the size of the single dots in the stimuli were randomly changed from trial to trial whereas their overall surface area was kept constant.

#### Habituation phase

Before starting the experiment, in order to let the fish acclimatise to the setup, in the two days preceding the experiment each fish was taken from the housing system and singly placed into the experimental tank. During this time, the fish had the possibility to get accustomed to the tank and in this way to reduce the potential stress related to the change of environment.

After this familiarization period, fish started the habituation phase. Each fish received 12 daily trials, divided in 3 sessions of 4 trials each (one session every three hours). In each trial, one panel depicting a precise numerosity (3 or 9 dots) was inserted in proximity to one of the two shorter sides of the tank. Thirty seconds after the stimulus was inserted, a morsel of food (1–1.2 mm size) was manually released in front of the panel. After the food was delivered, the stimulus remained in the tank for 2 minutes before being removed. The inter-trial time was 5 minutes; after this time, a new trial started on the opposite short tank side. Half of the subjects were habituated with the “3 dots” stimulus and the other half with the “9 dots” stimulus.

Since we wanted to exclude the possibility of the fish habituating to a particular visual pattern created by the dots’ position, we randomized the set of stimuli presentation. Thus, the spatial distribution and the size of each dot changed between trials, but the numerosity (3 or 9) and the cumulative surface area of the dots were kept constant. Fish were habituated following this procedure for four consecutive days. On the fifth day, fish performed only the first habituation session (4 trials). Subsequently, 5 hours later, fish underwent the dishabituation test. It was important to introduce such a time window between the last habituation trial and the dishabituation test in order to allow any IEG expression, associated with presentation of the stimuli during the habituation trials performed in the morning session, to return to a baseline level.

#### Dishabituation phase

The dishabituation phase consisted of a single trial in which the fish was exposed to a novel stimulus. The subjects were randomly subdivided in 5 different groups: a control group characterized by fish that had been presented with the familiar stimulus as in the habituation phase; a number change group in which the stimulus depicted a different number of items with respect to that of the habituation phase (i.e. from 3 to 9 or *vice versa* – change of number) but the same overall area; a shape change group in which the stimulus depicted a different shape but the same number and overall area; two size groups in which subjects were presented with a stimulus depicting the same number and shape as those of the familiar stimulus but a different size (increase: three times larger, or decrease: three times smaller). The dishabituation phase consisted of one single trial in which the dishabituation stimulus was inserted and left in the tank for 5 minutes. No food was provided to the fish during this phase. Thirty minutes after the dishabituation test, fish were sacrificed and their brains were dissected to quantify changes in the expression of the immediate early genes *c-fos* and *egr-1*.

As a behavioural measure, we analyzed the fish behaviour in the 30 seconds after the stimulus was shown, measuring the time spent in an area of 3 cm in proximity of the stimulus (see Fig. 1). In order to detect whether fish spent a different time in the area when the novel dishabituation stimulus was presented with respect to the habituation stimulus, a proportion of time was calculated comparing the dishabituation trial with the previous habituation trial session (the first of the four trials) performed on the same day.

### Tissue preparation: brain dissection, total RNA extraction

Thirty minutes after the dishabituation test, fish were sacrificed by putting them in a bath of ice-cold phosphate-buffered saline solution (PBS; Fisher Bioreagents, USA). Then, their brains were dissected and the expression of *c-fos* and *egr-1* was analyzed.

Telencephalon, thalamus, optic tectum, retina, cerebellum and medulla oblongata were collected and used for total RNA extraction. Total RNA was extracted using the QIAGEN RNeasy Mini Kit (QIAGEN, Germany) according to manufacturer’s instructions. Briefly, brain tissues were homogenized in lysis buffer, centrifuged and then loaded onto RNeasy spin columns. Samples were treated with DNase (RNase-Free DNase Set; QIAGEN, Germany) in order to prevent genomic DNA contamination. After a series of washes and centrifugations, total RNA was eluted from columns and quantified using the Nanodrop^TM^ spectrophotometer (Nanodrop^TM^ One^C^; ThermoFisher Scientific, USA). Reverse transcription was performed using the SuperScript^TM^ VILO^TM^ cDNA Synthesis Kit (Invitrogen, ThermoFisher Scientific, USA) according to manufacturer’s instructions.

### Quantitative/real-time polymerase chain reaction (qPCR)

Quantitative PCR (qPCR) experiments were performed in order to analyse the expression of the IEGs *c-fos* (NM_205569) and *egr-1* (NM_131248) and of the *18S* ribosomal RNA (*18S*) (NM_173234), which was used as reference gene. Specific primer pairs were commercially synthesized (Sigma-Aldrich/Merck; see Table 1). qPCR assays were performed in triplicate reactions using the PowerUp^TM^ SYBR^TM^ Green Master Mix (2X) and run in a CFX96^TM^ Real-Time System (Bio-Rad, USA). The ΔCq method was used for expression quantification^61–63^. Data were normalized on the expression of the *18* *S* reference gene (ΔCq) and the relative expression (to the reference gene) of each target was calculated.

**Table 1:** Primers used in this work^63–65^. Fw: forward primer, Rv: reverse primer.

Primer Name	Primer Sequence
c-fos Fw	GTATTACCCGCTCAACCAGAC
c-fos Rv	TCCAGTAACCCTCATTTTGGG
egr-1 Fw	AGTTTGATCACCTTGCTGGAG
egr-1 Rv	AACGGCCTGTGTAAGATATGG
18 s Fw	TCGCTAGTTGGCATCGTTTATG
18 s Rv	CGGAGGTTCGAAGACGATCA

### Statistical analyses

Statistical analyses on behaviour and reverse transcription qPCR (RT-qPCR) data were performed using the Statistical Package for the Social Sciences (IBM SPSS Statistics; IBM, USA). Behavioural data were analysed using non-parametric tests due to inhomogeneity of variances (Kruskal-Wallis analysis of variance plus Dunn’s post hoc tests with Bonferroni correction). Data for qPCR were analysed with a repeated measures analyses of variance checking for normality of the data (Shapiro-Wilk p > 0.05) and applying the Greenhouse-Geisser correction to adjust for the lack of sphericity. LSD post hoc tests with Bonferroni correction for multiple comparisons were used for pairwise comparisons.

### Ethical regulations

All husbandry and experimental procedures complied with the European Legislation for the Protection of Animals used for Scientific Purposes (Directive 2010/63/EU) and were approved by the Scientific Committee on Animal Health and Animal Welfare (Organismo Preposto al Benessere Animale, OPBA) of the University of Trento and by the Italian Ministry of Health (Protocol n. 893/2018-PR).

## Supplementary information

Supplementary Figure and Tables.

Data Availability.

## Data Availability

Data are available in a submitted Supplementary file.
