# Supplementary material for: Response to change in the number of visual stimuli in zebrafish:A behavioural and molecular study
Source: Sci Rep. 2020 Apr 1;10:5769. doi: 10.1038/s41598-020-62608-5 (PMC7113307; doi:10.1038/s41598-020-62608-5)
Supplement: Supplementary file 1 — Supplementary Figure and Tables. [file 41598_2020_62608_MOESM1_ESM.pdf]

Supplementary Materials of "Response to change in the number of visual stimuli in zebrafish: A behavioural and molecular study"

Andrea Messina[1,*], Davide Potrich[1], Ilaria Schiona[1], Valeria Anna Sovrano[1,2], Scott E. Fraser[3], Caroline H. Brennan[4], Giorgio Vallortigara[1]

[1] Center for Mind/Brain Sciences, University of Trento, Rovereto, Italy.

[2] Department of Psychology and Cognitive Science, University of Trento, Rovereto, Italy.

[3] Michelson Center for Convergent Bioscience, University of Southern California, Los Angeles, USA

[4] School of Biological and Chemical Sciences, Queen Mary University London, UK.

Supplementary Materials Figure 1: Proportion of time spent near the stimulus during dishabituation (comparing the dishabituation trial with the first of the four trials previously performed during the last habituation session) as a function of habituation conditions (with 3 or 9 dots) and test conditions [no change (familiar), change in number, change in shape, change in surface area (increase), change in surface area (decrease)].

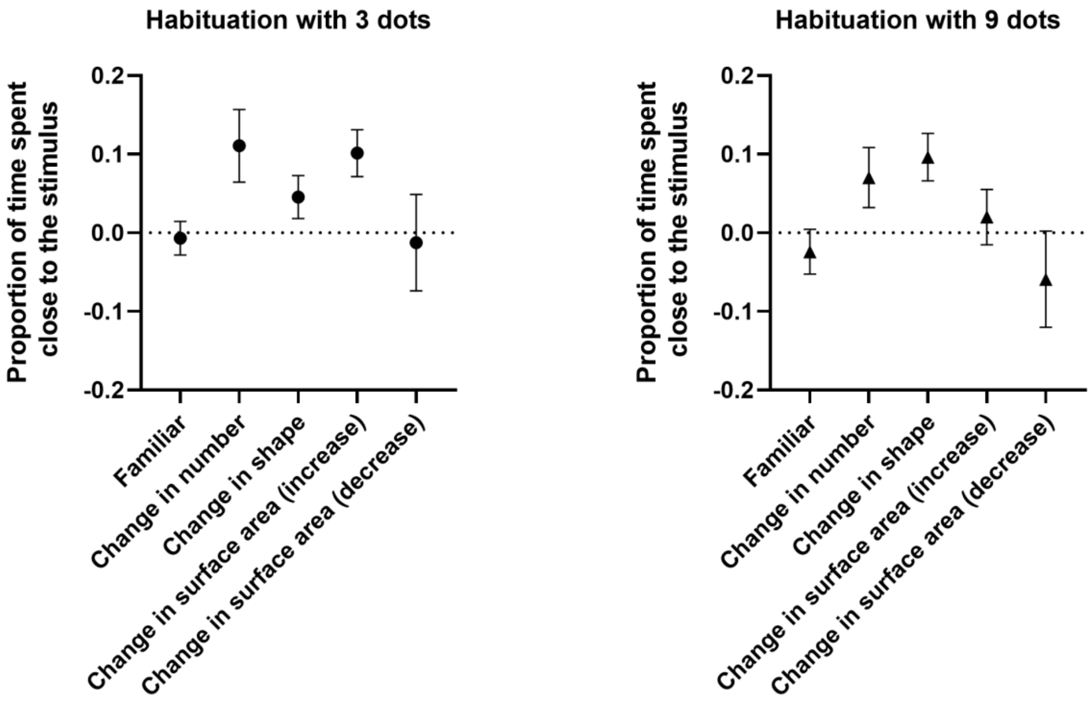

Supplementary Table 1: Overall analyses of variance (ANOVA) for *c-fos* and for *egr-1*, with habituation (habituation with 3 dots, habituation with 9 dots) and type of test (familiar, number, shape, surface area increase, surface area decrease) as between-subject factors, and brain areas (retina, optic tectum, thalamus, telencephalon, cerebellum, medulla oblongata) as a within-subject factor.

ANOVA			
c-fos	Main effect of Brain Area	F(4.221, 253.272)=53.493	p=0.0001
	Main effect of Habituation	F(1, 60)=5.062	p=0.028
	Main effect of Test	F(4, 60)=4.655	p=0.002
	Brain Area x Test interaction	F(16.885, 253.272)=2.605	p=0.001
	Habituation x Test interaction	F(4, 60)=14.255	p=0.0001
	Brain Area x Habituation interaction	F(4.221, 253.272)=3.774	p=0.005
	Brain Area x Test x Habituation interaction	F(16.885, 253.272)=33.228	p=0.0001
egr-1	Main effect of Brain Area	F(3.334, 200.028)=205.880	p=0.0001
	Main effect of Habituation	F(1, 60)=23.106	p=0.0001
	Main effect of Test	F(4, 60)=6.211	p=0.0001
	Brain Area x Test interaction	F(13.335, 200.028)=2.915	p=0.001
	Habituation x Test interaction	F(4, 60)=5.717	p=0.001
	Brain Area x Habituation interaction	F(3.334, 200.028)=3.663	p=0.011
	Brain Area x Test x Habituation interaction	F(13.335, 200.028)=1.934	p=0.027

Supplementary Table 2: Analyses of variance (ANOVA) for *c-fos* and for *egr-1* for the Retina, with habituation (habituation with 3 dots, habituation with 9 dots) and type of test (familiar, number, shape, surface area increase, surface area decrease) as between-subject factors.

RETINA			
c-fos	Main effect of Habituation	F(1, 60)=0.077	p=0.783
	Main effect of Test	F(4, 60)=1.643	p=0.0175
	Habituation x Test interaction	F(4, 60)=7.929	p=0.0001
egr-1	Main effect of Habituation	F(1, 60)=0.126	p=0.723
	Main effect of Test	F(4, 60)=2.981	p=0.026
	Habituation x Test interaction	F(4, 60)=13.541	p=0.0001

Supplementary Table 3: Analyses of variance (ANOVA) for *c-fos* and for *egr-1* for the Optic Tectum, with habituation (habituation with 3 dots, habituation with 9 dots) and type of test (familiar, number, shape, surface area increase, surface area decrease) as between-subject factors.

OPTIC TECTUM			
c-fos	Main effect of Habituation	F(1, 60)=0.852	p=0.360
	Main effect of Test	F(4, 60)=8.410	p=0.0001
	Habituation x Test interaction	F(4, 60)=2.146	p=0.086
egr-1	Main effect of Habituation	F(1, 60)=20.291	p=0.0001
	Main effect of Test	F(4, 60)=6.948	p=0.0001
	Habituation x Test interaction	F(4, 60)=4.243	p=0.004

Supplementary Table 4: Analyses of variance (ANOVA) for *c-fos* and for *egr-1* for the Thalamus, with habituation (habituation with 3 dots, habituation with 9 dots) and type of test (familiar, number, shape, surface area increase, surface area decrease) as between-subject factors.

THALAMUS			
c-fos	Main effect of Habituation	F(1, 60)=1.141	p=0.290
	Main effect of Test	F(4, 60)=6.329	p=0.0001
	Habituation x Test interaction	F(4, 60)=8.629	p=0.0001
egr-1	Main effect of Habituation	F(1, 60)=11.356	p=0.001
	Main effect of Test	F(4, 60)=4.369	p=0.004
	Habituation x Test interaction	F(4, 60)=2.190	p=0.081

Supplementary Table 5: Analyses of variance (ANOVA) for *c-fos* and for *egr-1* for the Telencephalon, with habituation (habituation with 3 dots, habituation with 9 dots) and type

of test (familiar, number, shape, surface area increase, surface area decrease) as between-subject factors.

TELENCEPHALON			
c-fos	Main effect of Habituation	F(1, 60)=7.930	p=0.007
	Main effect of Test	F(4, 60)=2.065	p=0.097
	Habituation x Test interaction	F(4, 60)=5.588	p=0.001
egr-1	Main effect of Habituation	F(1, 60)=15.969	p=0.0001
	Main effect of Test	F(4, 60)=2.124	p=0.089
	Habituation x Test interaction	F(4, 60)=6.144	p=0.0001

Supplementary Table 6: Analyses of variance (ANOVA) for *c-fos* and for *egr-1* for the Cerebellum, with habituation (habituation with 3 dots, habituation with 9 dots) and type of test (familiar, number, shape, surface area increase, surface area decrease) as between-subject factors.

CEREBELLUM			
c-fos	Main effect of Habituation	F(1, 60)=8.713	p=0.005
	Main effect of Test	F(4, 60)=1.025	p=0.402
	Habituation x Test interaction	F(4, 60)=4.037	p=0.006
egr-1	Main effect of Habituation	F(1, 60)=3.405	p=0.070
	Main effect of Test	F(4, 60)=3.616	p=0.010
	Habituation x Test interaction	F(4, 60)=1.726	p=0.156

Supplementary Table 7: Analyses of variance (ANOVA) for *c-fos* and for *egr-1* for the Medulla Oblongata, with habituation (habituation with 3 dots, habituation with 9 dots) and type of test (familiar, number, shape, surface area increase, surface area decrease) as between-subject factors.

MEDULLA OBLONGATA			
c-fos	Main effect of Habituation	F(1, 60)=0.090	p=0.765
	Main effect of Test	F(4, 60)=3.956	p=0.006
	Habituation x Test interaction	F(4, 60)=10.348	p=0.0001
egr-1	Main effect of Habituation	F(1, 60)=5.657	p=0.021
	Main effect of Test	F(4, 60)=0.561	p=0.692
	Habituation x Test interaction	F(4, 60)=2.440	p=0.056
